# Population-Based Prevalence of Antibiotic Residuals in Low, Moderate and High Malaria Endemicity Areas in Tanzania

**DOI:** 10.3390/antibiotics14020193

**Published:** 2025-02-13

**Authors:** Theopista Lotto, Joanna Gallay, Martin Zuakulu, Beatrice Ternon, Laurent Arthur Decosterd, Alexandra V. Kulinkina, Blaise Genton

**Affiliations:** 1 Swiss Tropical and Public Health Institute, Kreuzstrasse 2, 4123 Allschwil, Switzerland; alexandra.kulinkina@swisstph.ch; 2 Department of Public Health, University of Basel, 4001 Basel, Switzerland; 3 Ifakara Health Institute, Dar es Salaam P.O. Box 78373, Tanzania; martinzuakulu@hotmail.com; 4 Center for Primary Care and Public Health, 1010 Lausanne, Switzerland; joanna.gallay@unil.ch (J.G.); blaise.genton@chuv.ch (B.G.); 5 Laboratory of Clinical Pharmacology, Lausanne University Hospital, 1011 Lausanne, Switzerland; beatrice.ternon@chuv.ch (B.T.); laurentarthur.decosterd@chuv.ch (L.A.D.); 6 Center for Primary Care and Public Health, University of Lausanne, 1015 Lausanne, Switzerland

**Keywords:** antimicrobial resistance (AMR), community survey, dried blood spot (DBS), drug pressure, residual antibiotics, Tanzania

## Abstract

Background: Inappropriate antibiotic use drives antimicrobial resistance and remains a global concern. Evidence suggests antibiotic use may be higher among malaria-negative patients compared to malaria-positive ones, but uncertainty persists, particularly in regions with varying malaria prevalence. This study measured antibiotic residuals in three Tanzanian regions with varying malaria epidemiology and analyzed factors influencing their presence. Methods: A cross-sectional household survey was conducted in 2015, covering a population of 6000 individuals across three regions of Tanzania. Dried blood spot samples from a subset of participants were analyzed using broad-range tandem mass spectrometry to detect residual antibiotics. Risk factors associated with antibiotic presence, including household healthcare-seeking behaviors, malaria testing, and other relevant variables, were evaluated. Results: The overall prevalence of residual antibiotics in the study population was 14.4% (438/3036; 95% CI: 11.4–15.8%). Stratified by malaria transmission intensity, antibiotic prevalence was 17.2% (95% CI: 12.9–17.2%) in Mwanza (low), 14.6% (95% CI: 10.6–15.0%) in Mbeya (moderate), and 11.2% (95% CI: 7.9–11.6%) in Mtwara (high). Trimethoprim was the most frequently detected antibiotic (6.1%), followed by sulfamethoxazole (4.4%) and penicillin V (0.001%). Conclusions: Residual antibiotic prevalence did not directly correlate with malaria endemicity but was influenced by healthcare practices, including co-prescription of antibiotics and antimalarials. The higher antibiotic use in malaria-negative cases highlights the need for improved diagnostics to reduce unnecessary use and mitigate antimicrobial resistance in malaria-endemic areas.

## 1. Introduction

Tanzania has made notable strides in controlling malaria, with Malaria Indicator Survey reports showing a significant decline in prevalence among children under five years, from 15% in 2015 to 7.9% in 2022 [1]. This progress is largely attributed to a combination of vector control strategies [2], preventive therapies [3], and improved diagnostics, notably the use of malaria rapid diagnostic tests (mRDTs) [4]. However, research has shown that the use of mRDTs has led to increased antibiotic prescriptions for negative results due to insufficient guidance on non-malaria fevers and lack of diagnostic support for distinguishing viral and bacterial infections [5,6].

As malaria rates continue to decline, especially in low-endemic regions, the challenge of managing non-malarial fevers without over-relying on antibiotics is key in preventing antimicrobial resistance (AMR). In 2019, deaths from AMR worldwide surpassed those from both HIV/AIDS and malaria combined, highlighting AMR’s escalating impact on global health [7]. This crisis threatens to undermine decades of medical progress, making routine surgeries, cancer treatments, and even common infections far more dangerous [8]. The World Health Organization (WHO) has identified AMR as one of the top ten global health threats, calling for immediate action to combat the spread of resistant bacteria [9]. Such actions include monitoring antibiotic use in regions with different disease burdens, such as areas with varying levels of malaria endemicity, and strengthening antibiotic stewardship to ensure the responsible use of antimicrobials [10,11].

Previous studies have examined diagnostic practices and the use of antimalarial and antibiotic drugs in various malaria-endemic regions. D’Acremont et al. (2011) conducted a study in Dar es Salaam (a high-malaria area) to assess the impact of rapid diagnostic tests on antimalarial consumption [12]. Their findings revealed a significant reduction in antimalarial prescriptions, while also highlighting the frequent co-prescription of antibiotics and antimalarials, a practice linked to diagnostic challenges in managing febrile illnesses. Similarly, Gallay et al. (2018) found that 20.8% of blood samples in three Tanzanian regions with low, intermediate, and high malaria rates contained residual antimalarials, shedding light on the patterns of drug use and misuse, with recent fever history and living in areas with high malaria prevalence identified as key contextual factors [13]. Additionally, Lotto et al. (2024) focused specifically on children in both low- and high-malaria regions and reported a prevalence of 17.4%, with the highest rates observed in children under five years old [14].

Despite Tanzania’s progress in reducing malaria prevalence, the widespread use of mRDTs has inadvertently led to increased antibiotic prescriptions for individuals testing negative for malaria. While previous studies have explored diagnostic practices and drug use in high-malaria areas, little is known about antibiotic use trends across regions with varying malaria endemicities. Addressing this gap is important for developing antibiotic stewardship policies and mitigating the risk of AMR.

To contribute to this effort, this study examined the prevalence and associated factors of residual antibiotics in a previously underexamined context by leveraging data from the 2015 Antimalarial Concentration Study by Gallay et al. [13]. Using a subset of blood samples collected in 2015, this research investigated antibiotic exposure patterns across regions with different malaria endemicities. The results of this study will provide information about the patterns of antibiotic exposure, contributing to more effective antimicrobial stewardship and improved diagnostic practices in areas with varying malaria prevalence.

## 2. Materials and Methods

### 2.1. Study Design, Setting, and Population

A household cross-sectional survey, including interviews and dried blood spot (DBS) sample collection, was conducted after the rainy season. Laboratory analysis of the blood samples was performed to quantify residual antibiotics, which were then examined alongside individual and household-level variables collected during the survey. The survey was conducted between May and August 2015 in three Tanzanian regions with differing levels of malaria endemicity: Mtwara (20%) and Mwanza (15%), representing moderate to high endemicity, and the Mbeya-Songwe region, with a less than 1% prevalence, classified as low endemicity as per Tanzania’s National Bureau of Statistics [15]. Mtwara and Mwanza are characterized by tropical climates, whereas Mbeya–Songwe has a temperate climate [16]. In each region, both urban and rural councils were included in the survey.

### 2.2. Sample Size, Study Sampling and Data Collection

As a secondary analysis, a random selection of 3036 DBS samples from the main study [13] were analyzed from a pool of 6391 samples. The selection of the 3036 samples was made to achieve sufficient statistical power to detect a prevalence of 10% or higher of antibiotics found in the DBSs, thus ensuring the reliability and accuracy of the study’s findings (with a power of 80% and a confidence level of 95%), considering an estimated intracluster correlation coefficient of 0.0229. These calculations were performed using the epi.ssclus2estb function within the epiR package in R software (version 4.2.1).

The study selected one urban and two rural councils from each region, and within each council, three wards were randomly chosen proportionally to their population size. In each urban ward, four streets were randomly selected, while in each rural ward, two villages, as well as two sub-villages per village, were selected. From each street or sub-village, 20 households were randomly selected and up to six participants were randomly selected in each sampled household until a sample of 60 individuals per sub-village/street was reached, resulting in 240 individuals sampled per ward. Within each household, all individuals were given the opportunity to participate, excluding those with severe illnesses requiring immediate referral and those who were infants under three months of age. Interviews were conducted with a questionnaire in Swahili, starting with the head of the household. The questions included information on the time to the closest health facility and the closest pharmacy or drug retailer. Randomly selected family members were then asked about demographic information, any history of fever in the previous two weeks, health-seeking behavior, as well as history of antimalarial use in the previous months. Blood samples were collected from all participants using filter paper. To reach the sample size for the current sub-study, random sampling proportional to the number of samples collected in each region was employed, ensuring equal distribution across all study areas from the main study’s 6391 DBS samples. Further details on the sampling and data collection methods are provided elsewhere [17].

### 2.3. Laboratory Field Procedures

Capillary blood was collected on-site from all participants for direct mRDTs analysis (ParaHIT-f test, Span diagnostic Ltd., Surat, India, detecting HRP-2 antigens) and 4 drops were applied on filter paper cards (FTA DMPK-B cards, Whatman, GE Healthcare, Chicago, IL, USA) for the subsequent quantification of antimalarials and antibiotics in the laboratory. The DBS samples were dried at room temperature for at least two hours before being placed in a re-sealable plastic bag with desiccant and stored in a −10 °C freezer at the end of the day at the study site. Samples were then transferred to a −80 °C freezer at the Ifakara Health Institute within one month of collection and, finally, sent to the University Hospital of Lausanne, Switzerland, for analysis. All analytes were stable when stored for 24 h at room temperature and at 37 °C. Stability was also observed when replicating the study’s storage and transportation conditions. The 15 antibiotics quantified included amoxicillin, ampicillin, cloxacillin, penicillin G, penicillin V, ceftriaxone, cephalexin, trimethoprim, sulfamethoxazole, azithromycin, erythromycin, doxycycline, ciprofloxacin, metronidazole, and chloramphenicol.

### 2.4. Stability of Antibiotics in Dried Blood Spot

During DBS sample collection in Tanzania, all necessary precautions were taken to minimize the time the samples were exposed to room temperature. However, it was still essential to assess the stability of antibiotics under various storage conditions, including extreme environments that could be encountered during the study. To evaluate stability, quality control samples at three concentration levels (low, medium, and high) were applied to filter paper cards, with each concentration tested in triplicate on three separate cards (refer to Table S1). The prepared cards were then sealed in zip-lock bags containing two desiccants and placed in a box to protect them from light. The samples were stored at −80 °C (freezer), −20 °C (freezer), room temperature, or 37 °C with humidity (incubator). The stability of the antibiotics was analyzed after one and two weeks, with the results expressed as deviations from the initial concentrations recorded on day 0.

The findings indicated that all antibiotics remained stable for up to two weeks when stored at −80 °C and −20 °C. Interestingly, an apparent increase in concentration was observed for ciprofloxacin, doxycycline, sulfamethoxazole, chloramphenicol, and erythromycin, exceeding 15%. This could be attributed to the greater stability of antibiotics in DBS samples compared to whole blood calibrators, which were stored at −20 °C. The differences in calibration curves over time suggest that the perceived increase in DBS concentration may be due to the degradation of whole blood calibrators rather than an actual rise in drug content. This issue could be minimized by either preparing fresh calibrators for each analysis or storing them at −80 °C. In contrast, most antibiotics, except trimethoprim and sulfamethoxazole, exhibited significant degradation when stored at room temperature and 37 °C. Notably, β-lactam antibiotics, including amoxicillin, ceftriaxone, cephalexin, penicillin G, penicillin V, and cloxacillin, degraded substantially within just one week. This suggests that, while DBS samples generally enhance drug stability, they do not provide sufficient protection for highly sensitive antibiotics such as β-lactams.

### 2.5. DBS Sample Preparation and Analysis

The sample preparation began by extracting antibiotics from the dried blood spots. A 3 mm disk was punched from the DBS sample using 3 mm Uni-Core™ micro-punches and was extracted with a methanol solution containing isotopically labeled internal standards. The extraction process included sonication for 30 min and centrifugation at 13,000 rpm for 10 min to remove particulate matter. After centrifugation, the supernatant was diluted with an aqueous mobile phase (mobile phase A) and subjected to another round of centrifugation for 10 min. The resulting solution was then injected into the LC-MS/MS (Thermo Fisher Scientific TSQ Quantiva system, manufactured by Thermo Fisher Scientific, headquartered in Waltham, MA, USA), which used a Waters XSelect HSS T3 column (manufactured by Waters Corporation headquartered in Milford, MA, USA) for chromatographic separation. The system was maintained at a work temperature of 40 °C, optimizing conditions for analysis. Ionization was achieved under specific settings: spray voltage of +3500/−3200 V, sheath gas at 50 °C, auxiliary gas at 15 °C, and sweep gas at 0 °C. The ion transfer tube temperature was set to 350 °C, the vaporizer temperature to 40 °C, and the source height was adjusted to medium/high (M/H) to enhance the ionization efficiency. Data acquisition and quantification were controlled through Xcalibur 2.0.7 SP1 and LCQUAN 2.5.6 SP1 software.

Quantification was performed using a calibration curve, with corrections applied through the internal standards method. Stock solutions of antibiotics were stored at −80 °C and diluted to prepare calibration standards and quality control samples. Calibration standards were created by serial dilutions of the working solution to achieve target concentrations for each antibiotic. Each sample was diluted in 900 µL of whole hemolyzed blood to achieve the desired concentrations for analysis. The internal standards method ensured accurate quantification by accounting for variations in the analysis. The concentration of each antibiotic in the DBS samples was determined by comparing the sample’s response to the calibration curve, providing reliable and precise quantification. Each procedure was conducted according to the standard operating procedures.

### 2.6. Validation of LC-MS/MS Method for the Quantification of Antibiotics

The validation of the LC-MS/MS method for this study was performed by assessing various parameters specific to the analysis of different antibiotics (Table S2). The limits of detection (LLOD) and limits of quantification (LLOQ) were established for all antibiotics in the study. These values were essential in determining the lowest concentration at which each antibiotic could be reliably detected and quantified, as detailed in the table. The extraction recovery efficiency (extraction ER %) was evaluated for each compound at different concentrations, with results varying across antibiotics and concentrations. The overall extraction efficiency ranged from moderate to high, with certain compounds demonstrating extraction recoveries above 90%. The coefficient of variation (CV %) for extraction efficiency and analysis efficiency (analysis ER %) were calculated to assess the precision and reproducibility of the method. Most antibiotics exhibited low CVs, indicating the good precision of the method. Matrix effects (ME %) were also assessed to examine the impact of sample matrices on the accuracy of quantification. The matrix effects were calculated for different antibiotics and showed varying levels, with certain compounds demonstrating a higher degree of matrix interference than others.

### 2.7. Statistical Analysis

Questionnaires were designed using Open Data Kit, and data was stored on the ODK Aggregate data repository at the end of each survey day. Data cleaning and analysis were conducted using R (version 4.2.1). All analysis variables were summarized using common summary statistics appropriate to the variable type (Table 1). A univariable and multivariable mixed-effects logistic regression model (lme4 package) was used to identify factors associated with the presence of antibiotics in the blood, and odds ratios (ORs), their 95% confidence intervals (CIs), and *p*-values are reported. Ward was included as a random effect in all analyses to account for clustering of observations. Statistical significance was determined by a *p*-value < 0.05.

## 3. Results

### 3.1. Population Characteristics from the Household Surveys

The analysis included 3036 individuals, with 2030 individuals (66.9%) residing in rural and 1003 (33.1%) in urban settings. The median age of study subjects was 17 years, ranging from 3 months to 100 years. Females accounted for 55.1% of the participants, while the predominant age group (39%) was 25 years and above. Approximately half (57.6%) of the participants lived less than one hour from government health facilities and 16.3% reported having experienced fever in the past two weeks. Among the subjects surveyed, 16.9% had positive malaria rapid diagnostic tests (Table 2).

### 3.2. Prevalence of Antibiotics in the Blood of the Surveyed Population

**Figure 1:**
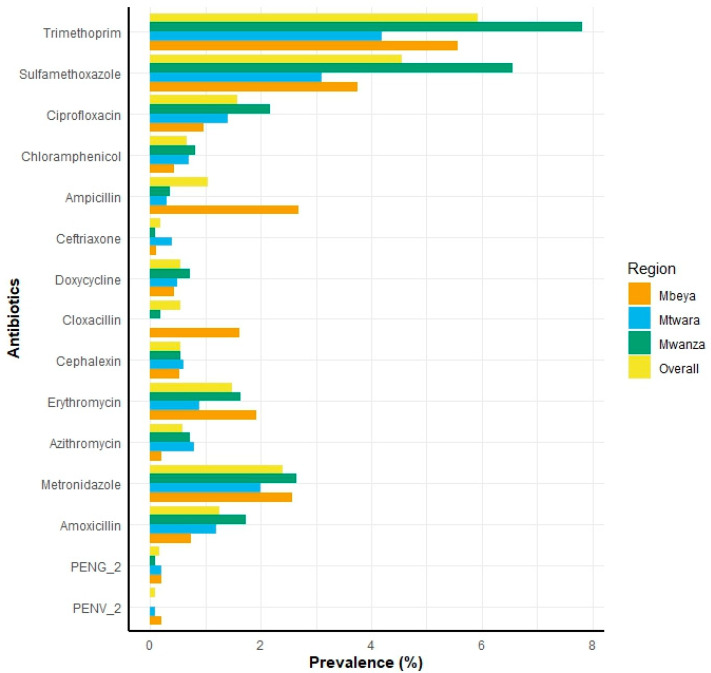
Distribution of detected antibiotics across three regions, alongside the overall prevalence rates in Tanzania, 2015. PENG-2 refers to penicillin G (also known as benzylpenicillin), and PENV_2 refers to penicillin V (also known as phenoxymethylpenicillin).

The overall prevalence of residual antibiotics in the DBS of the studied population was 14.4%. Among these, the most prevalent antibiotic was trimethoprim, with a prevalence of 5.9%, followed by sulfamethoxazole (4.5%) and metronidazole (2.4%) (Figure 1). The proportions of individuals with antibiotics found in their blood were as follows: 8.7% had one antibiotic, 4.7% had two antibiotics, 1.0% had three antibiotics and 0.1% had four antibiotics. The overall prevalence of detected antibiotics in blood varied across regions: in Mwanza, it reached 17.2%, while in Mbeya; it was 14.6%, and 11.2% in Mtwara.

**Figure 2:**
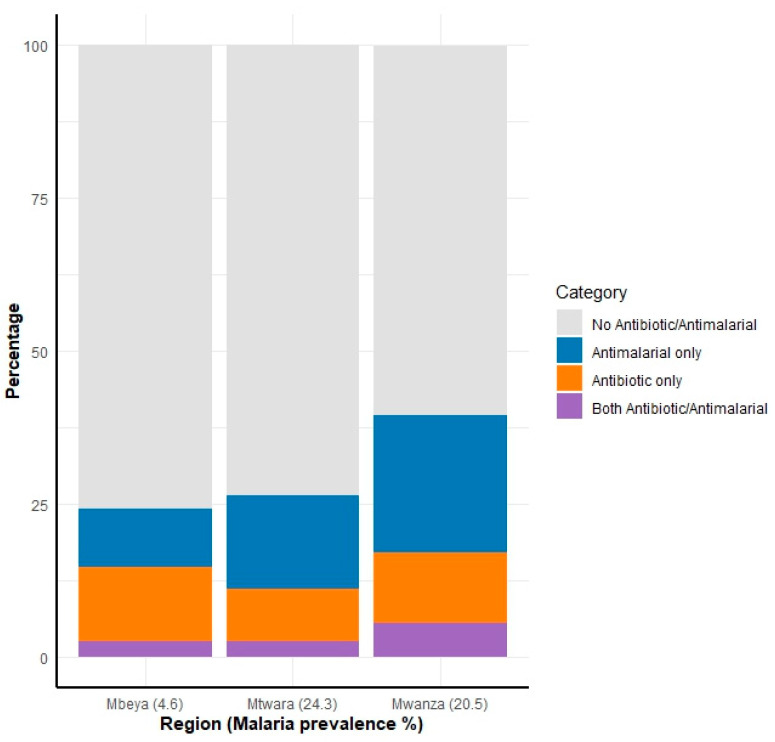
Distribution of antibiotics and antimalarials detected in blood samples across the three studied regions in Tanzania, 2025. From the left, the first stacked bar represents the Mbeya region, which recorded the lowest levels of residual antimicrobials (antibiotics and antimalarials), followed by the Mtwara and Mwanza regions, with Mwanza showing the highest levels of residual drugs detected. The regions are arranged in ascending order based on the detected residual drug levels. The numbers in brackets next to the region names on the *x* axis indicate malaria prevalence, as determined by mRDT during the household survey.

The analysis of the studied regions shows varying proportions of individuals with antibiotics, antimalarials, or both in their blood (Figure 2). Antimalarial detection rates were highest in Mwanza (22.4%), followed by Mtwara (15.2%) and Mbeya (9.6%). These detection rates correspond to a malaria prevalence of 20.5% in Mwanza, 24.3% in Mtwara, and 4.6% in Mbeya based on mRDT results obtained during the survey period.

The prevalence of residual antibiotics varied significantly across age groups: 17.1% in children aged 0–4 years, 16.0% in adults aged 25 and older, 12.6% in young adults aged 15–24 and 11.9% in children aged 5–14. Individuals living within one hour of a government healthcare facility had a prevalence of 15.3%. In contrast, those residing near non-government health facilities showed a prevalence of 17.8% for the same distance category. Additionally, the prevalence among individuals with a negative malaria test was notably higher at 15.2%, compared to 9.6% for those with a positive test result. Statistically significant differences among these groups are highlighted in Table 3.

### 3.3. Factors Associated with the Presence of Antibiotics

Several factors were associated with higher odds of having residual antibiotics in blood samples. Females were more likely to have antibiotics in their blood compared to males (OR = 1.30, 95% CI: 1.05–1.61). Individuals who tested negative for malaria had higher odds of residual antibiotics compared to those who tested positive (OR = 1.46, 95% CI: 1.04–2.06). Additionally, individuals with antimalarial drugs in their blood were more likely to have antibiotics detected than those without antimalarial drugs (OR = 1.37, 95% CI: 1.06–1.77). Conversely, certain demographic and travel-related factors were associated with lower odds of residual antibiotics. Individuals aged 5–14 years demonstrated 44% lower odds (OR = 0.66, 95% CI: 0.48–0.91) and those 15–24 years had 43% lower odds (OR = 0.67, 95% CI: 0.46–0.98) of having antibiotics in their blood as compared to children aged 0–4 years. Additionally, individuals who had a travel time to non-governmental healthcare facilities of more than 1 h had lower odds (OR = 0.61, 95% CI: 0.42–0.88). No significant associations were found for the other variables (Table 3).

## 4. Discussion

This study investigates the prevalence and predictors of antibiotics detected in the DBS among the Tanzanian population across regions with varying malaria transmission rates. Using a highly sensitive LC-MS/MS laboratory method, the study accurately quantified residual antibiotics. In the surveyed regions, 14% of participants had detectable residual antibiotics, with trimethoprim and sulfamethoxazole being the most prevalent, aligning with findings from previous research [14].

### 4.1. Regional Variations in Detected Antibiotic Residuals

The study revealed regional variations in the prevalence of antibiotic residues: Mwanza (17.2%), Mbeya (14.6%) and Mtwara (11.2%). These differences may be attributed to multiple factors influencing antibiotic use patterns, as outlined in Table S3.

Climate and disease burden significantly affect antibiotic use [18,19]. Mwanza’s warm (28.5 °C) and relatively dry climate (1035.6 mm rainfall) favors vector-borne diseases, contributing to a malaria prevalence of 20.5%, which is lower than Mtwara’s (24.3%) but higher than Mbeya’s (4.6%) [16,20]. Mwanza also recorded the highest antimalarial detection rate (22.4%), likely due to malaria treatment efforts, which may include antibiotics for secondary infections [21]. In contrast, Mtwara, with warmer (31 °C) and rainier (1220.2 mm) conditions, faces optimal malaria transmission conditions, yet its lower antimalarial detection rate (15.2%) may be due to limited healthcare access [20,22,23,24]. Mbeya’s cooler climate (24.7 °C) and lower malaria prevalence (4.6%) reduce malaria transmission, but its 9.6% antimalarial detection rate suggests possible overuse due to presumptive treatment [17].

Healthcare access further influences antibiotic and antimalarial use [15]. Mwanza, with 30 hospitals, 72 health centers, and 385 dispensaries, has relatively better healthcare availability, though 45.4% of the population still faces accessibility challenges, potentially leading to self-medication [25,26,27]. Mtwara, with the lowest healthcare infrastructure (12 hospitals, 47 health centers, and 280 dispensaries), sees 59.4% of residents struggling to access care, which may explain the lower antibiotic detection rates despite a high malaria burden [27,28]. Informal drug markets or traditional medicine may supplement formal treatment [29]. Mbeya’s healthcare comprises 20 hospitals, 56 health centers, and 356 dispensaries, with 42.4% of the population reporting difficulties accessing care [27]. While its lower malaria prevalence likely contributes to reduced antibiotic use, a higher HIV burden (9.3%) may lead to increased antibiotic prescriptions for opportunistic infections, as recommended by the Tanzanian National Guidelines for the Management of HIV and AIDS [30].

Socioeconomic factors such as poverty, household size, and literacy also influence antibiotic consumption [31,32]. Mwanza’s high population density (371.8 people/km^2^) and poverty levels (1,184,188 individuals) likely drive increased antibiotic use due to household disease transmission [32,33]. Mtwara’s smaller household sizes (3.8), delayed healthcare-seeking, and reliance on traditional medicine may explain the lower antibiotic detection rates despite its high malaria burden [32,33]. In contrast, Mbeya’s lower poverty levels (439,447 individuals) and higher literacy rate (78.7%) may contribute to more rational antibiotic use, though its HIV burden sustains demand [32,33]. Study conducted in Kenya, Tanzania, and Uganda found that individuals facing financial barriers and limited healthcare access were more likely to misuse antibiotics through self-medication and non-adherence [31]. Similarly, in Pakistan, lower socioeconomic status was linked to reduced medical consultations and incomplete antibiotic courses, increasing the risk of misuse [34]. In Northern Ghana, larger household sizes were associated with higher antibiotic use, likely due to increased transmission within crowded living conditions [35]. Additionally, a study among Tanzanian parents showed that lower education levels were linked to poor antibiotic practices in children [36].

### 4.2. Comparison with Other Studies in Similar Malaria-Endemic Regions

A comparison with studies from other malaria-endemic regions, such as Cameroon, Ghana, and other parts of Africa, could provide context to understand antibiotic-prescribing behaviors. For instance, research from Cameroon found that 36.7% of primary healthcare prescriptions included antibiotics, often due to diagnostic uncertainty and limited laboratory access [37]. This highlights a common challenge faced by healthcare providers in malaria-endemic settings, where distinguishing between bacterial and non-bacterial infections remains difficult.

Similarly, a study in Ghana revealed that despite a 50% decline in malaria prevalence, antibiotic use remained high due to persistent challenges in distinguishing febrile illnesses [10]. This trend is further supported by findings from the Democratic Republic of the Congo, where patients with uncomplicated malaria were frequently co-prescribed antibiotics [38]. This indicates a growing pattern of concurrent medication use, often in the absence of bacterial infections confirmed by diagnostic tests.

Further reinforcing this trend, research in Sudan highlighted that even eight years after the introduction of artemisinin-based combination therapies, patients continued to receive antibiotics alongside antimalarials, despite no confirmed bacterial infections [39]. One of the key drivers of this practice is the fear of misdiagnosis, as malaria symptoms can overlap with bacterial infections [40]. In resource-limited settings, where diagnostic tools are scarce, clinicians often adopt a precautionary approach, prescribing both antimalarials and antibiotics to minimize the risk of under-treatment.

Likewise, another study highlighted the frequent co-prescription of antibiotics with antimalarial drugs, indicating a need to address irrational prescribing practices [41]. Moreover, a study reported a significant increase in antibiotic prescriptions, rising from 49% before the introduction of mRDTs to 72% after their implementation (RR = 1.47, 95% CI 1.37–1.59) [12]. These patterns suggest a need to better understand prescribing behaviors in malaria-endemic regions, particularly the factors driving concurrent antibiotic and antimalarial use, to inform more targeted interventions that optimize treatment decisions and reduce unnecessary antibiotic exposure. Moreover, future studies should explore the effectiveness of policies aimed at reducing unnecessary antibiotic prescriptions, such as stricter adherence to mRDT results or clinician training programs.

### 4.3. Factors Associated with Antibiotics Detection in Blood Samples

Several factors were associated with higher odds of residual antibiotics in blood samples, including being female, testing negative for malaria, and having an antimalarial in the bloodstream.

While prescribing patterns contribute to antibiotic exposure, the availability and distribution of these medications also play a crucial role. Beyond clinical settings, dispensing practices influence access to antibiotics, often enabling misuse and overuse. A study conducted in the Mwanza region in Nyamagana, Ilemela, and Sengerema districts, as well as in other regions, revealed several factors contributing to poor dispensing practices, including pressure from customers, the profit-making orientation of drug dispensers, and customers’ economic circumstances [42,43]. A study also identified a widespread willingness to dispense antibiotics without a prescription in community pharmacies and accredited drug-dispensing outlets in the area [44]. These findings emphasize the importance of addressing accurate diagnoses at the level of the informal sector and implementing careful drug use to combat inappropriate dispensing practices and antibiotic reliance in the region.

In addition to dispensing practices, demographic factors also shape patterns of antibiotic exposure. Notably, women exhibited a higher prevalence of residual antibiotics (16.1%) compared to men (12.4%), a finding that aligns with a meta-analysis conducted in nine high-income countries [45]. A similar pattern was observed in Bangladesh, where women were more likely to receive antibiotic prescriptions than men (OR = 4.04, 95% CI 1.55, 10.55) [46]. This discrepancy can be attributed to the fact that women are more prone to infections, such as urinary or gynecological conditions, which often result in higher antibiotic prescriptions [47]. While gender differences contribute to antibiotic exposure, age and recent illness history also play a role. This study observed lower odds of antibiotic residuals among individuals aged 10–24 and those who reported no illness in the preceding two weeks. This may be due to the lower morbidity rates from infectious diseases in these age groups compared to children under five years of age [48,49].

This study possesses several scientific strengths. Firstly, it employed LC-MS/MS technology, which allowed for the precise measurement of antibiotic concentrations in blood samples. Additionally, the inclusion of a representative sample of the general population enabled a comprehensive assessment of antibiotic exposure across diverse demographic groups. Through capturing not only prescribed antibiotics from healthcare settings but also those obtained through self-medication, the study provides a more accurate estimate of overall antibiotic exposure. Furthermore, the analysis of a broad range of commonly used antibiotics strengthens the validity of the findings, offering valuable insights into the multiple pathways of antibiotic exposure in the population.

This study has several limitations that should be considered when interpreting its findings. First, although efforts were made to ensure proportional representation across regions, the focus on one urban and two rural councils per region may not fully capture the diversity of antibiotic exposure across all geographic and demographic contexts. Additionally, the selection of specific councils may introduce selection bias, potentially over- or underestimating antibiotic exposure patterns in certain subpopulations. Future studies could address this by incorporating a broader range of regions with diverse demographic and epidemiological profiles. Additionally, key variables such as socioeconomic status, education level, and marital status, which have been shown to influence antibiotic exposure in previous studies [50,51,52], were not collected in this study, potentially limiting the understanding of contextual factors affecting the findings. Incorporating clinical diagnostic data, such as recent diagnoses of bacterial infections, could have provided deeper insights into the appropriateness of antibiotic use. Future studies should consider integrating clinical and prescription data to enhance interpretability. Furthermore, while the method used to detect antibiotics in DBS samples was robust, certain antibiotics or metabolites may not have been detectable, potentially underestimating the prevalence of exposure. The findings are also context-specific to the regions studied, and caution should be exercised when generalizing results to other settings with differing healthcare systems, antibiotic prescribing practices, or population characteristics. Lastly, the temporal context of the study, including factors such as seasonal variations in antibiotic use or public health interventions during the data collection period, may limit the broader applicability of the results.

## 5. Conclusions

This study identifies a complex relationship between antibiotics and malaria endemicity settings. While the prevalence of residual antibiotics does not directly align with malaria transmission rates, the findings suggest that healthcare provider practices, particularly the co-prescription of antibiotics and antimalarials, play a significant role. The higher odds of antibiotic detection among malaria-negative individuals also point to the need for improved diagnostic capacities to reduce unnecessary antibiotic use. These insights are important for designing targeted interventions that balance effective malaria management with the judicious use of antibiotics, ultimately mitigating the risk of antimicrobial resistance in malaria-endemic regions.

## Figures and Tables

**Table 1:** Summary of the variables used in the study.

Variable	Type	Definition
Outcome		
Antibiotics in blood	Binary	Presence of any antibiotic(s) in the blood equal or above the limits of quantification
Predictors		
Age (years)	Ordinal	Participant’s age, categorized as 0–4, 5–14, 15–24, or 25+
Sex	Categorical	Categorized as male or female
Time to the nearest government healthcare facilities	Ordinal	Time required to reach nearby government health facility, categorized as <15 min, 15 min–1 h, ≥1 h, or unknown
Time to the nearest nongovernment hospital	Categorical	Time required to reach nearby nongovernment health facility, categorized as <15 min, 15 min to <1 h, ≥1 h, or unknown
Time to the nearest medical shop	Categorical	Time required to reach nearby medical shop, categorized as <15 min, 15 min to <1 h, ≥1 h, or unknown
mRDT results on site	Categorical	Rapid malaria diagnostic test taken during the survey categorized as positive, negative, not valid, or missing
History of fever in the past 14 days	Categorical	Self-reported fever in past 14 days prior to the survey, categorized as yes, no, or does not know
Sought care for this fever	Categorical	Whether care was sought in case of fever, categorized as yes, no, or does not know
Time taken to travel to the provider	Categorical	Time required to reach healthcare provider, categorized as <15 min, 15 min to <1 h, ≥1 h, or unknown
Malaria blood test performed	Categorical	Malaria diagnostic test performed when seeking care, categorized as?
Malaria test results	Categorical	Reported result of the malaria diagnostic test performed when seeking care, categorized as positive, negative, or not valid

Variables analyzed in the study, detailing their types and definitions. Abbreviation: mRDT = malaria rapid diagnostic test.

**Table 2:** Demographic characteristics of study participants (*N* = 3036).

Variable	Frequency (*n*)	Percentage (%)
Sex		
Male	1359	44.80
Female	1674	55.10
Missing	3	0.10
Age (Years)		
0–4	531	17.50
5–14	839	27.60
15–24	428	14.10
25+	1185	39.00
Time to Government Health Facility		
<15 min	811	26.70
15 min to <1 h	1748	57.60
1 h or more	386	12.70
Missing	3	0.10
Time to Non-Government Health Facility		
<15 min	474	15.60
15 min to <1 h	1041	34.30
1 h or more	431	14.20
Missing	3	0.10
Time to Medicine Shops		
<15 min	1353	44.60
<1 h	1262	41.60
≥1 h	418	8.50
Missing	3	0.10
mRDT Result		
Positive	512	16.90
Negative	2500	82.20
Missing	24	2
Had Fever in the Previous 2 Weeks		
Yes	494	16.30
No	2539	83.50
Missing	3	0.10
Had Antimalarial in Blood		
Yes	599	19.7
No	2435	80.2
Missing	3	0.1
Regions		
Mbeya	933	30.80
Mtwara	1000	32.90
Mwanza	1100	36.20
Missing	3	0.10

**Table 3:** Results of the univariable and multivariable mixed-effects logistic regression models explaining the presence of residual antibiotics in the blood samples of the study participants (*N* = 3036).

Variables	Total Participants *N* (%)	Participants with Antibiotics in the Blood				
		*N* (%)	Univariable Analysis		Multivariable Analysis	
			OR (95% CI) *	*p*-Value	aOR (95% CI) *	*p*-Value
Total	3036	438 (14.4)				
Sex						
Male	1359 (44.8)	168 (12.4)	Ref		1	
Female	1674 (55.1)	269 (16.1)	1.33 (1.08–1.64)	**0.008**	1.30 (1.05–1.61)	**0.017**
Missing	3 (0.1)	1 (33.3)				
Age (Years)						
0–4	531 (17.5)	91 (17.1)	Ref	**--**		
5–14	839 (27.6)	100 (11.9)	0.64 (0.47–0.88)	**0.006**	0.66 (0.48–0.91)	**0.011**
15–24	428 (14.1)	54 (12.6)	0.65 (0.45–0.94)	**0.022**	0.67 (0.46–0.98)	**0.039**
25+	1185 (39.0)	190 (16.0)	0.94 (0.71–1.25)	0.682	0.93 (0.70–1.23)	0.598
Time to Government Health Facility						
<15 min	811 (26.7)	115 (14.2)	Ref	--	--	--
15 min to <1 h	1748 (57.6)	268 (15.3)	1.13 (0.88–1.46)	0.329	1.15 (0.87–1.51)	0.322
1 h or more	386 (12.7)	54 (11.4)	0.75 (0.49–1.13)	0.171	0.91 (0.59–1.39)	0.659
Missing	3 (2)	1 (33.3)				
Non-Government HF time						
<15 min	474 (15.6)	92 (19.4)	Ref	--	--	--
15 min to <1 h	1041 (34.3)	185 (17.8)	1.01 (0.74–1.37)	0.967	1.03 (0.74–1.44)	0.869
1 h or more	431 (14.2)	160 (10.5)	0.60 (0.40–0.92)	**0.018**	0.61 (0.42–0.88)	**0.024**
Missing	3 (2)	1 (33.3)				
Time to medicine shops						
<15 min	1353 (44.6)	218 (16.1)	Ref	--	--	--
<1 h	1262 (41.6)	177 (14.0)	0.98 (0.77–1.25)	0.891	0.95 (0.73–1.24)	0.721
≥1 h	418 (8.5)	42 (10.0)	0.69 (0.44–1.10)	0.118	0.89 (0.59–1.36)	0.600
Missing	3 (2)	1 (33.3)				
mRDT result						
Positive	512 (16.9)	49 (9.6)	Ref	--	--	--
Negative	2500 (82.2)	381 (15.2)	1.57 (1.13–2.18)	**0.007**	1.46 (1.04–2.06)	**0.029**
Missing	24 (2)	8 (33.3)				
Had fever in the previous 2 weeks						
Yes	494 (16.3)	86 (17.4)	Ref	--	--	--
No	2539 (83.5)	351 (13.8)	0.75 (0.57–0.98)	**0.034**	0.78 (0.59–1.03)	0.083
Missing	3 (2)	1 (33.3)				
Had antimalarial in blood						
Yes	599 (19.7)	111 (18.5)	1.48 (1.16–1.89)	**0.002**	1.37 (1.06–1.77)	**0.014**
No	2435 (80.2)	327 (13.4)	--	**--**	--	**--**
Missing	3 (0.1)	0				
Regions						
Mbeya	933 (30.8)	136 (14.6)		--	--	--
Mtwara	1000 (32.9)	112 (11.2)	0.79 (0.51–1.22)	0.291	0.87 (0.58–1.28)	0.476
Mwanza	1100 (36.2)	189 (17.2)	1.30 (0.85–1.98)	0.222	1.17 (0.81–1.70)	0.397
Missing	3 (0.1)	1 (33.3)				

* Odds ratios (ORs) or adjusted odds ratios (aORs) and 95% confidence intervals (CIs) for fixed effects are shown. All models also included cluster as a random effect. Statistical significance was determined by a *p*-value < 0.05, and all statistically significant values are bolded. “Ref” stands for reference group used to denote the baseline or comparison group in the regression analysis.

## Data Availability

All data generated or analyzed during this study are available from the corresponding author upon reasonable request.

## Supplementary Materials

The following supporting information can be downloaded at: https://www.mdpi.com/article/10.3390/antibiotics14020193/s1. Table S1: Results of the stability assay. Table S2: Limits of detection and quantification, as well as the matrix effect, extraction recovery, and precision efficiency assessment for antibiotics. Table S3: Demographic data comparison across the Mwanza, Mtwara, and Mbeya regions.
