# Supplementary material for: Population-Based Prevalence of Antibiotic Residuals in Low, Moderate and High Malaria Endemicity Areas in Tanzania
Source: Antibiotics (Basel). 2025 Feb 13;14(2):193. doi: 10.3390/antibiotics14020193 (PMC11851362; doi:10.3390/antibiotics14020193)
Supplement: Supplementary file 1 [file antibiotics-14-00193-s001.zip › antibiotics-3444830-supplementary.pdf]

Supplemental Information

Table S1. Results of the stability assay

Table S2. Limits of detection and quantification, Matrix effect, Extraction Recovery and Precision Efficiency assessment for antibiotics

Table S3. Demographics Data Comparison across Mwanza, Mtwara, and Mbeya Regions

Table S1: Results of the stability assay

**Antibiotics**		**Amoxicillin**			**Metronidazole**			**Ceftriaxone**			**Trimethoprim**		
Nominal concentration [ng/ml]		450	1800	18000	600	2400	24000	1200	4800	48000	30	120	1200
0		0%	0%	0%	0%	0%	0%	0%	0%	0%	0%	0%	0%
week	-80%	-5%	0%	9%	0%	3%	1%	26%	47%	7%	5%	11%	15%
	-20%	-4%	1%	12%	2%	-3%	6%	27%	43%	5%	5%	8%	15%
1	Room T^0	-93%	-84%	-75%	-16%	-5%	-1%	-5%	-10%	-8%	-2%	-3%	6%
week	37^0C(+humidity)	-97%	-100	-100%	8%	3%	17%	-81%	-56%	-77%	14	12%	26%
	-80^0 C	22%	35%	50%	33%	31%	34%	49%	40%	34%	25	33%	46%
	-20^0C	33%	23%	36%	19%	11%	25%	36%	32%	28%	18	23%	39%
2	Room T^0	-94%	-92%	-90%	10%	4%	11%	-22%	-20%	-26%	21	16%	25%
week	37^0C(+humidity)	-97%	-99%	-100%	8%	21%	17%	-81%	-73%	-77%	14	26%	26%
**Antibiotics**		**Cephalexin**			**Ciprofloxacin**			**Doxycycline**			**Sulfamethoxazole**		
Nominal concentration [ng/ml]		1500	6000	60000	21	84	840	600	2400	24000	375	3000	30000
0		0%	0%	0%	0%	0%	0%	0%	0%	0%	0%	0%	0%
week	-80%	1%	24%	14%	-2%	36%	8%	12%	19%	16%	18%	28%	23%
	-20%	3%	15%	14%	-3%	48%	7%	11%	23%	12%	28%	21%	21%
1	Room T^0	-44%	-35%	-38%	-29%	5%	0%	-13%	3%	-4%	-29	4%	4%
week	37^0C(+humidity)	-89%	-76%	-90%	-51%	-17%	-31%	-60%	-47%	-47%	-24	-24	-25
	-80^0 C	18%	36%	40%	11%	49%	65%	40%	33%	23%	41%	45%	41%
	-20^0C	17%	21%	34%	1%	31%	42%	17%	38%	21%	30%	24%	37%
2	Room T^0	-41%	-43%	-43%	-15%	1%	14%	-11%	-11%	-1%	-1%	0%	1%
week	37^0C(+humidity)	-89%	-87%	-90%	-51%	-21%	-31%	-60%	-52%	-47%	-24	-24	-25
**Antibiotics**		**Chloramphenicol**			**Erythromycin**			**Penicillin G**			**Penicillin V**		
Nominal concentration [ng/ml]		1200	4800	48000	150	600	6000	1500	6000	60000	1500	6000	60000
0		0%	0%	0%	0%	0%	0%	0%	0%	0%	0%	0%	0%
week	-80%	24%	40%	26%	13%	22%	25%	5%	25%	12%	14%	34%	34%
	-20%	28%	31%	27%	16%	22%	24%	5%	22%	7%	21%	33%	27%
1	Room T^0	1%	8%	-1%	-7%	1%	-1%	-67%	-63%	-67%	-75	-69	-71
week	37^0C(+humidity)	-56%	-20%	-59%	-41%	-26%	-43%	-99%	-100	-100%	-95	-99	-100
	-80^0 C	17%	46%	48%	15%	40%	47%	6%	31%	40%	16%	28%	58%
	-20^0C	30%	31%	46%	7%	32%	37%	11%	22%	28%	19%	29%	43%
2	Room T^0	7%	15%	13%	3%	19%	13%	-86%	-87%	-89%	-93	-92	-92
week	37^0C(+humidity)	-56%	-47%	-59%	-41%	-31%	-43%	-99%	-100	-100%	-95	-98	-100
**Antibiotics**		**Cloxacillin**											
Nominal concentration [ng/ml]		1200	4800	48000									
0		0%	0%	0%									
week	-80%	12%	26%	24%									
	-20%	8%	25%	17%									

1 week	Room T^0	-70%	-64%	-65%
	37^0C(+humidity)	-100	-100	-100%
	-80^0 C	25%	40%	31%
	-20^0C	25%	25%	27%
2 week	Room T^0	-88%	-87%	-89%
	37^0C(+humidity)	-100	-100	-100%

The percentage change in antibiotic concentration at different time points (0, 1, and 2 weeks) under four storage conditions: room temperature (RT), 37°C with humidity, −20°C, and −80°C. The nominal concentrations of each antibiotic are provided, and stability is represented by the percentage change in concentration.

Table S2: Limits of detection and quantification (LLOD - LLOQ), Matrix effect (ME), Extraction Recovery (ER) and Precision Efficiency (PE) assessment for antibiotics.

	LLOD - LLOQ	$C_{Nominal}$	Mean peak area			Mean peak-area ratio			ME (%)	ME (%)	Extraction ER %	CV (%)	Analysis ER (%)	CV (%)	PE %	CV (%)
Compound	(ng/mL)	(ng/ml)	A* (n=3)	B (n=6)	C (n=5)	A2*	B2	C2	B/A	B2/A2	C/B		C2/B2		C/A	
Metronidazole	25 - 50	150	3232602	3082628	2177669	9.666	9.007	6.700	95.4	93.2	70.6	4.4	74.4	7.0	67.4	4.2
		400	8384923	7488321	5941888	25.571	22.822	19.252	89.3	89.3	79.3		84.4		70.9	
		4000	83746420	80093511	54696713	254.779	248.111	187.393	95.6	97.4	68.3		75.5		65.3	
Amoxycillin	20 – 20	60	47966	46113	32454	0.143	0.135	0.100	96.1	93.9	70.4	13.8	74.1	11.1	67.7	5.9
		160	119671	122772	88023	0.365	0.374	0.285	102.6	102.5	71.7		76.2		73.6	
		1600	1069545	1286863	717306	3.254	3.986	2.458	120.3	122.5	55.7		61.6		67.1	
Ceftriaxone	25 - 50	150	583444	638090	170422	0.138	0.160	0.087	109.4	115.9	26.7	4.8	54.4	8.4	29.2	3.6
		400	1553557	1597763	459577	0.398	0.433	0.262	102.8	108.6	28.8		60.6		29.6	
		4000	16233644	17407396	4301267	3.952	4.717	2.423	107.2	119.3	24.7		51.4		26.5	
Ampicillin	17.5 - 37.5	113	332222	327492	229992	0.089	0.093	0.066	98.6	103.7	70.2	9.2	71.2	11.2	69.2	4.3
		300	885231	869013	624289	0.252	0.267	0.199	98.2	106.2	71.8		74.6		70.5	
		3000	9090909	9849628	5480584	2.510	3.075	1.840	108.3	122.5	55.6		59.8		60.3	
Trimetoprim	7.5 - 15	45	738489	694566	531813	0.204	0.202	0.152	94.1	98.9	76.6	10.0	75.3	8.1	72.0	6.4
		120	1708884	1710399	1353077	0.513	0.545	0.434	100.1	106.2	79.1		79.7		79.2	
		1200	17000447	18072900	11689935	4.931	5.934	4.018	106.3	120.3	64.7		67.7		68.8	
Cephalexin	25 - 25	75	170299	189204	112470	0.052	0.061	0.041	111.1	117.1	59.4	7.1	67.7	10.2	66.0	2.8
		200	458354	450816	291063	0.144	0.152	0.120	98.4	105.1	64.6		78.8		63.5	
		2000	4563260	4932513	2501081	1.421	1.714	1.119	108.1	120.6	50.7		65.3		54.8	
Ciprofloxacine	10 - 10	15	246142	242819	146847	0.094	0.100	0.079	98.6	106.7	60.5	6.0	78.6	10.6	59.7	7.4
		40	630762	588929	368661	0.259	0.268	0.213	93.4	103.3	62.6		79.8		58.4	
		400	6335802	6648349	3311170	2.524	2.993	2.010	104.9	118.6	49.8		67.1		52.3	
Azithromycine	10 - 10	30	74737	72953	56691	0.210	0.211	0.165	97.6	100.4	77.7	6.1	77.8	10.8	75.9	7.5
		80	193279	192107	152176	0.547	0.576	0.447	99.4	105.4	79.2		77.6		78.7	
		800	1968617	2158539	1330690	5.243	6.214	4.033	109.6	118.5	61.6		64.9		67.6	
Sulfamethoxazole	37.5 - 75	225	1275800	1322969	839356	1.462	1.584	0.998	103.7	108.3	63.4	5.9	63.0	11.0	65.8	7.9
		600	3399686	3404692	2280531	4.024	4.355	2.877	100.1	108.2	67.0		66.1		67.1	

		6000	35041739	37692476	20853557	40.119	47.344	27.933	107.6	118.0	55.3		59.0		59.5	
Doxycycline	50 - 100	300	1065379	1076427	547189	0.107	0.116	0.070	101.0	108.8	50.8	6.1	60.4	10.6	51.4	7.5
		800	2710287	2603269	1318520	0.280	0.293	0.186	96.1	104.8	50.6		63.4		48.6	
		8000	28631263	29103242	12158644	2.709	3.131	1.753	101.6	115.6	41.8		56.0		42.5	
Chloramphenicol	50 - 50	150	65698	65038	49778	0.129	0.137	0.103	99.0	105.8	76.5	6.4	75.1	7.9	75.8	2.9
		400	179310	174080	132401	0.385	0.401	0.302	97.1	104.3	76.1		75.3		73.8	
		4000	1730515	1777945	1160348	3.451	4.017	2.777	102.7	116.4	65.3		69.1		67.1	
Erythromycine	15 - 15	45	196121	207018	160092	0.589	0.631	0.474	105.6	107.1	77.3	6.8	75.1	8.7	81.6	3.3
		120	515528	535978	414257	1.603	1.743	1.306	104.0	108.7	77.3		74.9		80.4	
		1200	5065637	5607147	3528960	15.590	18.391	12.036	110.7	118.0	62.9		65.4		69.7	
Penicilline G	25 - 50	150	253039	259697	188955	0.269	0.279	0.218	102.6	103.6	72.8	13.4	78.2	9.3	74.7	3.6
		400	688799	726159	539553	0.759	0.798	0.631	105.4	105.1	74.3		79.1		78.3	
		4000	7141579	8208961	4756143	7.559	8.980	5.851	114.9	118.8	57.9		65.1		66.6	
Penicilline V	50 - 100	300	313939	315215	227290	0.334	0.339	0.263	100.4	101.4	72.1	17.1	77.5	9.6	72.4	3.8
		800	859892	896575	650339	0.948	0.985	0.761	104.3	104.0	72.5		77.2		75.6	
		8000	9175436	10381275	5805269	9.712	11.357	7.141	113.1	116.9	55.9		62.9		63.3	
Cloxacillin	25 - 50	150	100130	96136	75776	0.167	0.166	0.134	96.0	99.4	78.8	17.4	80.9	9.3	75.7	0.9
		400	271374	267615	202462	0.466	0.484	0.382	98.6	103.8	75.7		78.8		74.6	
		4000	2726732	2947366	1745380	4.037	4.820	3.268	108.1	119.4	59.2		67.8		64.0	

**A = Matrix-free solution of analytes and I.S. in MeOH + 1%FA. A2 = internal standard-normalization using ratios of analyte peak areas to the corresponding I.S. peak area*

B = Blank DBS extracts samples spiked with antibiotic and I.S. after extraction. B2 = internal standard-normalization using ratios of analyte peak areas to the corresponding I.S. peak area

C = Complete procedure. C2 = internal standard-normalization using ratios of analyte peak areas to the corresponding I.S. peak area

Table S3: Demographics Data Comparison Across Mwanza, Mtwara, and Mbeya Regions

**Factor**	**References**	**Regions**		
		**Mwanza**	**Mtwara**	**Mbeya**
Population[a]	[33]	3,532,378	1,424,083	2,070,412
Area (km^2)	[16]	~ 9500	~16,710	~37,754
Population density (per sq. km)[b]	--	371.8	85.2	54.8
Average annual rainfall (mm)	[16]	1035.6	1220.2	1164.9
Average annual temperature (°C)	[16]	28.5	31	24.7
Percentage literate	[32]	78	75.5	78.7
Distribution of Basic Poor People	[32]	1,184,188	494,534	439,447
Average household size	[32]	5.8	3.8	3.7
Number of operating health facilities (hospitals/health centers/dispensaries)[c]	[27]	30/72/385	12/47/280	20/56/356
Problems in accessing health care due to distance (%)	[15]	45.4	59.4	42.4
Acute Respiratory Infections prevalence	[15]	4.3	4.8	2.6
Self-reported prevalence of diarrhea in the last 2 weeks among under five years (%)	[15]	11.7	18.3	13.9
HIV Prevalence Among Adults Aged 15 Years and Older	[15]	7.2%	2.0%	9.3%

[a]*Population was calculated by extrapolating village-level population of 2012 with the council level growth rate, per annum for the 2002-2012 intra-censal period, up to year 2015.*

[b]*Calculated by dividing population by area.*

[c]*The number of operating health facilities (hospitals, health centers, and dispensaries) was obtained from the health facility registry website. The figures presented exclude other healthcare facilities such as laboratories, pharmacies, and clinics*
